# Antifibrotic Effects of the Dual CCR2/CCR5 Antagonist Cenicriviroc in Animal Models of Liver and Kidney Fibrosis

**DOI:** 10.1371/journal.pone.0158156

**Published:** 2016-06-27

**Authors:** Eric Lefebvre, Graeme Moyle, Ran Reshef, Lee P. Richman, Melanie Thompson, Feng Hong, Hsin-l Chou, Taishi Hashiguchi, Craig Plato, Dominic Poulin, Toni Richards, Hiroyuki Yoneyama, Helen Jenkins, Grushenka Wolfgang, Scott L. Friedman

**Affiliations:** 1 Tobira Therapeutics, Inc., South San Francisco, CA, United States of America; 2 Chelsea and Westminster Hospital, London, United Kingdom; 3 Columbia Center for Translational Immunology, Columbia University Medical Center, New York, NY, United States of America; 4 Department of Medicine, University of Pennsylvania Perelman School of Medicine, Philadelphia, PA, United States of America; 5 AIDS Research Consortium of Atlanta, Atlanta, GA, United States of America; 6 Division of Liver Diseases, Icahn School of Medicine at Mount Sinai, New York, NY, United States of America; 7 Stelic Institute & Co., Inc., Tokyo, Japan; 8 Plato BioPharma, Inc., Westminster, CO, United States of America; 9 Charles River Laboratories Montreal ULC, Montreal, QC, Canada; 10 Independent Consultant, Kailua-Kona, HI, United States of America; Institute of Medical Research A Lanari-IDIM, University of Buenos Aires-National Council of Scientific and Technological Research (CONICET), ARGENTINA

## Abstract

**Background & Aims:**

Interactions between C-C chemokine receptor types 2 (CCR2) and 5 (CCR5) and their ligands, including CCL2 and CCL5, mediate fibrogenesis by promoting monocyte/macrophage recruitment and tissue infiltration, as well as hepatic stellate cell activation. Cenicriviroc (CVC) is an oral, dual CCR2/CCR5 antagonist with nanomolar potency against both receptors. CVC’s anti-inflammatory and antifibrotic effects were evaluated in a range of preclinical models of inflammation and fibrosis.

**Methods:**

Monocyte/macrophage recruitment was assessed *in vivo* in a mouse model of thioglycollate-induced peritonitis. CCL2-induced chemotaxis was evaluated *ex vivo* on mouse monocytes. CVC’s antifibrotic effects were evaluated in a thioacetamide-induced rat model of liver fibrosis and mouse models of diet-induced non-alcoholic steatohepatitis (NASH) and renal fibrosis. Study assessments included body and liver/kidney weight, liver function test, liver/kidney morphology and collagen deposition, fibrogenic gene and protein expression, and pharmacokinetic analyses.

**Results:**

CVC significantly reduced monocyte/macrophage recruitment *in vivo* at doses ≥20 mg/kg/day (*p* < 0.05). At these doses, CVC showed antifibrotic effects, with significant reductions in collagen deposition (*p* < 0.05), and collagen type 1 protein and mRNA expression across the three animal models of fibrosis. In the NASH model, CVC significantly reduced the non-alcoholic fatty liver disease activity score (*p* < 0.05 *vs*. controls). CVC treatment had no notable effect on body or liver/kidney weight.

**Conclusions:**

CVC displayed potent anti-inflammatory and antifibrotic activity in a range of animal fibrosis models, supporting human testing for fibrotic diseases. Further experimental studies are needed to clarify the underlying mechanisms of CVC’s antifibrotic effects. A Phase 2b study in adults with NASH and liver fibrosis is fully enrolled (CENTAUR Study 652-2-203; NCT02217475).

## Introduction

Fibrosis results from a sustained inflammatory response to chronic organ injury and is characterized by the deposition of extracellular matrix proteins, including collagen types 1 and 3 [1]. Hepatic fibrosis is associated with chronic liver disease, a significant global burden that contributes to cirrhosis and hepatocellular carcinoma [2]. Likewise, renal fibrosis is a common manifestation of chronic kidney disease [3], associated with elevated risks of morbidity and mortality [4]. Effective, well-tolerated antifibrotic and anti-inflammatory pharmacotherapies that can be integrated into current disease-management approaches are urgently needed.

The inflammatory response to hepatocyte injury plays a key role in hepatic fibrogenesis and involves recruitment of bone marrow-derived monocytes and macrophages to the site of injury, which is triggered by the activation of resident macrophages (i.e. Kupffer cells [KCs]) [1]. In turn, infiltrating monocytes/macrophages amplify this immune response by producing inflammatory cytokines and chemokines, which further promote recruitment of inflammatory cells and upregulate the activation of hepatic stellate cells (HSCs) [1,5]. Fibrogenic cytokines (e.g. transforming growth factor-beta [TGF-beta]), produced by activated macrophages, promote transdifferentiation of HSCs into myofibroblasts, which are the primary source of scar-forming matrix proteins, including fibrillary collagen types 1 and 3, and the contractile protein alpha-smooth muscle actin (alpha-SMA) [1,6–8].

Recruitment of extra-hepatic inflammatory cells to the site of hepatic injury is largely mediated by interactions between chemokines and their receptors. Monocytes, KCs and HSCs can express C-C chemokine receptor types 2 (CCR2) and 5 (CCR5) on their surface [9–12]. Increasing evidence implicates CCR2/CCR5 and their ligands (including C-C chemokine ligand type 2 [CCL2, aka monocyte chemotactic protein-1 (MCP-1)] and type 5 [CCL5, aka *Regulated on Activation*, *Normal T-cell Expressed and Secreted (RANTES)*]), in the pathogenesis of liver fibrosis through promotion of monocyte/macrophage recruitment and tissue infiltration, and HSC activation following liver injury [9–15]. Hepatocytes, KCs and infiltrating monocytes/macrophages are the main sources of TGF-beta, a major fibrogenic cytokine promoting collagen production by activated HSCs [12]. Additional evidence substantiates the roles of CCR2/CCR5 and their ligands in renal fibrosis [16–20]. Thus, CCR2 and CCR5 have become attractive targets for antifibrotic therapy.

Cenicriviroc (CVC) is a novel, oral, once-daily (QD) dual CCR2/CCR5 antagonist with nanomolar potency, and a long plasma half-life (30–40 hours in humans) [21–24]. It has a favorable safety profile and was well tolerated in approximately 600 subjects, including those with mild or moderate hepatic impairment (Child–Pugh A and B) [25,26]. CVC is currently under evaluation in a Phase 2b study in 289 adults with non-alcoholic steatohepatitis (NASH) and liver fibrosis, for which it received Fast Track Designation by the Food and Drug Administration (CENTAUR Study 652-2-203; NCT02217475) [27]. An *ex vivo* study of human peripheral blood mononuclear cells found that CVC leads to receptor occupancies of ~98% for CCR2 on monocytes (at 6 nmol/L) and ≥90% for CCR5 on CD4^+^ and CD8^+^ T-cells (at 3.1 and 2.3 nmol/L, respectively) [28]. As a shorter half-life (~2 hours in mice) and a lower potency have been observed for CVC in rodents *versus* humans, this was considered in dose selection for disease models. An *ex vivo* study conducted on mouse monocytes and macrophages showed that CVC concentrations of 250 nmol/L or higher achieve >87% CCR2/CCR5 occupancy in these cells [29,30]. Collectively, these findings suggest that rodent models are well suited to evaluate the anti-inflammatory and antifibrotic properties of CVC, resulting from effective CCR2/CCR5 blockade.

A number of *in vitro* and *in vivo* models of fibrosis are commonly used to assess recruitment of inflammatory cells and antifibrotic activity of therapeutic agents [31–33]. Multiple models of fibrosis allow assessment of the broad effect of an antifibrotic agent across species and organs, and reduce the likelihood that efficacy is restricted to one model. Here we provide evidence for the antifibrotic effects of CVC, as demonstrated in models that have evaluated: (1) the *ex vivo* and *in vivo* effects of CVC on recruitment/migration of monocytes/macrophages; and (2) the *in vivo* antifibrotic effects of CVC in liver and kidney fibrosis.

## Materials and Methods

All animal procedures were approved by each institution’s animal care and use committee (IACUC), and were conducted in accordance with national guidelines. CVC is cenicriviroc mesylate, provided by Tobira Therapeutics, Inc., USA. The vehicle control used in all *in vivo* studies was 0.5% [w/v] methylcellulose + 1% Tween^®^-80 (pH ~1.3).

### Effect of CVC on recruitment/migration of monocytes/macrophages

#### *In vivo* mouse model of peritonitis

A murine thioglycollate (TG)-induced model of peritonitis, where acute inflammation induced by intraperitoneal (IP) injection of TG results in a rapid increase in monocyte/macrophage migration into the peritoneal cavity [34], was employed to assess the effects of CVC on cell recruitment *in vivo*. The protocol was approved by the IACUC of Charles River Laboratories Preclinical Services, Montreal (PCS-MTL). The care and use of animals was conducted in accordance with the guidelines of the US National Research Council and the Canadian Council on Animal Care.

Male C57BL/6 mice (n = 44; 8–10 weeks of age; Charles River Laboratories, Canada) were allocated to receive treatments via oral gavage (PO) on Days 1–5 in the following groups: non-disease control, vehicle control twice daily (BID), CVC 5 mg/kg/day (CVC5) BID, CVC 20 mg/kg/day (CVC20) BID, CVC 100 mg/kg/day (CVC100) BID, CVC20 QD, and positive control dexamethasone (corticosteroid known to reduce inflammation in a variety of animal models) 1 mg/kg QD (S1 Table). On Day 4, peritonitis was induced via IP injection of TG 3.85% (1 mL/animal) 2 hours post-dose in all groups except non-disease controls. Study endpoints included: peritoneal lavage cell counts and pharmacokinetic (PK) evaluation. Animals were sacrificed 48 hours post-TG injection by isoflurane inhalation, and peritoneal lavage and blood samples (0.7 mL) were collected. Differential cell counts were assessed in peritoneal lavage samples using an Advia^®^ Hematology System (Siemens Healthcare Diagnostics, USA) with multispecies software and an analysis software designed for mouse peritoneal fluid on Advia^®^ 120 (LabThruPut, USA). A 0.3 mL aliquot of the blood sample was processed to plasma for PK analysis.

#### *Ex vivo* migration of mouse monocytes

The protocol was approved by the IACUC of the University of Pennsylvania (protocol number 804755) and animals were maintained according to the National Institutes of Health (NIH) guidelines. Animals were euthanized by CO_2_ inhalation followed by cervical dislocation.

Mouse monocyte migration in response to CVC treatment was assessed *ex vivo* in triplicate. TG was injected intraperitoneally into male C57BL/6 mice (n = 3; 8–10 weeks of age; Jackson Laboratory, USA) and activated macrophages were collected 48 hours later by peritoneal lavage. Chemotaxis was assayed using a Transwell^®^ Chamber (Costar, USA) with a 5 μm-pore size polycarbonate filter, as previously described [35]. Briefly, cells were incubated for 2 hours in the presence of 1 nM CCL2 and/or 1 μM CVC (dissolved in dimethyl sulfoxide with 0.5% acetic acid and diluted 1:1000 with serum-free Roswell Park Memorial Institute-1640 medium and 0.5% bovine serum albumin). Cells were harvested from the lower compartment and analyzed by flow cytometry to enumerate F4/80^+^CD11b^+^ macrophages using a 3-laser BD FACSCanto™ (BD Biosciences, Canada). Results were analyzed using FlowJo software (Tree Star Inc., USA).

### Antifibrotic effects of CVC in animal models of fibrosis

#### Rat model of thioacetamide (TAA)-induced liver fibrosis (TAA model)

The TAA model is commonly used for the evaluation of treatment at various stages of disease, from inflammation to cirrhosis [36]. The protocol was approved by the Mount Sinai IACUC (approval number: LA12-00318) and animals were maintained according to the NIH guidelines. Anesthesia was performed with 1–5% isoflurane through inhalation; surgery was terminal.

Using male Sprague-Dawley rats (n = 72, 10–12 weeks of age; Harlan Laboratories, USA), fibrosis was induced by IP administration of TAA at a dose of 150 mg/kg three times per week for 8 weeks. Rats (n = 4–8/group) received vehicle control, CVC 30 mg/kg/day (CVC30) or CVC100 QD PO during Weeks 0–8 (early intervention), Weeks 4–8 (established fibrosis) or Weeks 8–12 (cirrhosis reversal) and were sacrificed at Weeks 8, 8 or 12, respectively (S1 Table). Study endpoints included: body and liver weights, liver biochemistry (e.g. serum alanine and aspartate aminotransferase [ALT/AST]), extracellular matrix protein expression in liver tissue (collagen type 1 and alpha-SMA), mRNA expression (collagen type 1, alpha-SMA, beta-platelet-derived growth factor-beta receptor, TGF beta-receptor, matrix metalloproteinase 2, tissue inhibitor of metalloproteinases 1 [TIMP1] and 2 [TIMP2]) and liver morphology. For liver-function testing, plasma samples were obtained from blood collected from the vena cava. Livers were sectioned and fixed in 3.7% formalin, embedded in paraffin, cut at 4 mm thickness and stained with hematoxylin and eosin (H&E) for histological examination.

#### Mouse model of diet-induced NASH (NASH model)

The protocol was approved by Stelic IACUC (approval number: RP-131). All animals were housed and cared for in accordance with the Japanese Pharmacological Society Guidelines for Animal Use.

NASH was induced in male C57BL/6 mice (Charles River Laboratories, Japan) via subcutaneous injection of 200 μg of streptozotocin 2 days post-birth (causing mild islet inflammation and islet destruction) plus a high-fat diet (57 kcal% fat) from 4 weeks of age (sequentially causing fatty changes to the liver, NASH and fibrosis) [37]. From Weeks 6 to 9, three groups (n = 9/group) received vehicle control, CVC20 or CVC100 BID PO (S1 Table). At Week 9, six animals per group were sacrificed for assessment of liver fibrosis and Non-Alcoholic Fatty Liver Disease (NAFLD) Activity Score (NAS). The animals were sacrificed by exsanguination through direct cardiac puncture under ether anesthesia. Study endpoints included: body and liver weights, plasma biochemistry (e.g. ALT and CCL2), extracellular matrix protein in liver tissue (hydroxyproline content), mRNA expression (collagen type 1, tumor necrosis factor-alpha, MCP-1, TIMP1) and histopathological analyses. Blood samples were collected and plasma samples derived for biochemistry assessments. Sections were cut from paraffin blocks of liver tissue, prefixed in Bouin’s solution and stained with H&E for histological examination.

#### Mouse model of unilateral ureter obstruction (UUO)-induced renal fibrosis (UUO model)

Unilateral ureter obstruction is a commonly used experimental model of kidney injury, in which interstitial inflammation, tubular cell injury/death and fibrosis, ensue [32]. The protocol was approved by Plato BioPharma, Inc. IACUC (PBI; approval number 2013–04). The care and use of animals was conducted in accordance with the NIH Guide for the Care and Use of Laboratory Animals (2010), Action against Medical Accidents Guidelines on Euthanasia (2007), Office of Laboratory Animal Welfare Institutional Animal Care and Use Committee Guidebook (2002), Public Health Service Policy on Humane Care and Use of Laboratory Animals (2002), PBI standard operating procedures on a) moribund sacrifice, b) vivarium maintenance and animal care, and c) animal handling and dosing. Anesthesia was performed by isoflurane injection; animals were euthanized by diaphragm laceration followed by heart laceration while under isoflurane anesthesia.

Male CD-1 mice (n = 51; 7–8 weeks of age; Charles River Laboratories, USA) were allocated to weight-matched treatment groups on Day -1 (1 day prior to either sham [one group] or permanent right UUO surgery [five groups] via aseptic laparotomy). From Days -1 to 4, mice received phosphate-buffered saline (PBS) IP QD, apart from a positive-control group, which received 1D11 (anti-TGF-beta_1_ antibody) 3 mg/kg IP QD. From Days 0 to 5, mice received vehicle control (sham surgery, UUO-control and UUO+positive-control groups) or CVC 7 mg/kg/day (CVC7), CVC20 or CVC100 PO BID (S1 Table). The CVC100 group was terminated due to poor clinical condition (6/9 animals died and 3/9 euthanized prior to end of study). Of note, a similar dose of CVC100 administered for 3 weeks was well tolerated in the mouse NASH model; therefore, it is plausible that the laparotomy and UUO surgery in combination with CVC may have contributed to the loss of animals. Study endpoints included: body and kidney weights, mRNA expression (TGF-beta, connective tissue growth factor, MCP-1, alpha-SMA, collagen 1a1, fibronectin-1 and collagen 3a1), extracellular matrix protein in renal cortical tissue (hydroxyproline content) and histological analyses. Blood and tissue samples were collected from anesthetized mice 4 hours post-dose on Day 5, prior to sacrifice. A mid-transverse section of the right obstructed kidney was collected for histological analysis.

### Outcome measures in animal models of fibrosis

#### Body and liver/kidney weights

Body weight (measured before and during treatment) and sacrificed animal body and liver/kidney/spleen weights were recorded.

#### Plasma biochemistry

In the TAA model, serum ALT/AST levels were measured using VITROS^®^ 5,1 FS (Ortho Clinical Diagnostics, USA), with plasma diluted with VITROS^®^ 7% bovine serum albumin if needed. In the NASH model, plasma ALT levels were measured by FUJI DRI-CHEM 7000 (Fujifilm, Japan) and plasma CCL2 concentration quantified by the mouse CCL2/JE/MCP-1 immunoassay kit (R&D, USA).

#### Liver or obstructed kidney morphology

Collagen deposition (the extent of fibrosis) was visualized in liver/kidney sections with picrosirius red staining. In the TAA model, collagen quantification was performed using computerized Life Science morphometry system (BIOQUANT, USA) on a total of 36 images per animal at 100x magnification (four picrosirius red-stained slides per animal, with nine images taken randomly per slide).

In the NASH model, bright field images of picrosirius red-stained sections were captured around the central vein using a digital camera (DFC280; Leica, Germany) at 200x magnification; the ‘positive’ areas in five fields/sections were measured using ImageJ software (National Institutes of Health, USA). Perivascular areas were subtracted from the total positive areas for each field (modified fibrosis area). The NAS, a histological tool developed to assess disease severity in humans, was assessed in a blinded fashion and calculated according to Kleiner’s criteria on H&E-stained sections [38]. It is based on the semi-quantification of steatosis, lobular inflammation and hepatocellular ballooning.

In the UUO model, ten images/depth/kidney were assessed in a blinded fashion using Axio Imager.A2 (Zeiss, USA) light microscopy (at 200x magnification to enable 60–70% sampling of renal cortical area) and quantified by a composite Collagen Volume Fraction (CVF [% total area imaged]) score expressed as the average positive stain across three anatomically distinct (200–250 μM apart) tissue sections, or depths, from the obstructed kidney.

#### Immunohistochemistry

In the NASH model, liver sections fixed in acetone were incubated with anti-F4/80 antibody (BMA Biomedicals, Switzerland) to assess inflammation. Double immunohistochemical analyses were performed with anti-CD206 (RayBiotec, USA) or anti-CD16/32 (BD Biosciences, USA) antibodies. Cells were counted and the M1/M2 polarization ratio was calculated as mean percentage of F4/80+CD16/32+ cells/mean percentage of F4/80+CD206+ cells.

#### Extracellular matrix proteins

Extracellular matrix protein content in tissue samples was measured by evaluating collagen type 1 and alpha-SMA protein expression in the TAA model and hydroxyproline content in the NASH and UUO models.

In the TAA model, total protein was extracted from liver cells and expression levels of collagen type 1 and alpha-SMA were assessed by western blotting. Protein expression levels were normalized to the reference protein (glyceraldehyde-3-phosphate dehydrogenase [GAPDH] or calnexin).

In the NASH model, frozen liver samples were subjected to an alkaline-acid hydrolysis, then centrifuged, and the supernatant collected. Hydroxyproline content was quantified against a hydroxyproline standard curve, with a BCA protein assay kit (Thermo Fisher Scientific, USA) used to normalize the calculated hydroxyproline values.

In the UUO model, frozen renal cortical tissue biopsies were hydrolyzed and centrifuged, and the supernatant was analyzed by OD absorbance at 560 nM on a SpectraMax^®^ 190 (Molecular Devices, USA). Standard curves for conversion of ODs to concentrations were generated using linear regression and sample concentrations were determined using SoftMax^®^ Pro5 software (Molecular Devices, USA).

#### Gene expression of fibrotic or inflammatory biomarkers

In the hepatic-fibrosis models, RNA was extracted from liver tissues and purified, and 1 μg of total mRNA was reverse-transcribed into complementary DNA. Expression levels were determined by quantitative polymerase chain reaction (PCR) (iQ™ SYBR^®^ Green Supermix [Bio-Rad Laboratories, USA] on the LightCycler^®^ 480 Real-Time PCR System [Roche, Switzerland]) in the TAA model at Week 8 (early intervention and established fibrosis) and Week 12 (cirrhosis reversal), and by real-time PCR (PCR Dice^®^ and SYBR^®^ Premix Ex Taq™ [Takara Bio Inc., Japan]) at Week 9 in the NASH model.

In the UUO model, mRNA expression levels in renal cortical tissue were evaluated using the QuantiGene^®^ Plex 2.0 profiling platform (Affymetrix, USA).

Relative mRNA expression levels were normalized to the following reference genes: TAA model, GAPDH; NASH model, 36B4 (gene symbol: Rplp0); UUO model, hypoxanthine phosphoribosyltransferase.

### PK analysis in the TG-induced model of peritonitis and the UUO model

CVC plasma levels (minimum and maximum) were determined by KCAS Bioanalytical Services, USA, using a validated liquid chromatography-tandem mass spectrometry (LC/MS/MS) plasma method (50 μL assay, range 10.0−1920.0 ng/mL).

### Statistical analysis

In all studies, statistical significance level was set as *p* < 0.05.

#### Recruitment/migration of monocytes/macrophages

In the peritonitis mouse model, one-way analysis of variance with post-hoc Dunnett’s test was performed, using GraphPad Prism^®^ (GraphPad Software, Inc., USA), on differential cell counts obtained from lavage in treatment groups *versus* the vehicle-control group. For the *ex vivo* migration of mouse monocytes, statistical significance was determined by a Student’s *t* test.

#### Animal models of fibrosis

Data are presented as ±standard error of the mean (SEM) in the TAA model. Statistical significance was determined by the two-tailed Student’s *t* test.

In the NASH model, data are expressed as mean ±standard deviation (SD). Statistical analyses were performed using the Bonferroni multiple comparison test (GraphPad Prism^®^ 4 software).

In the UUO model, data are expressed as mean ±SEM. All statistical analyses were performed using GraphPad Prism^®^ 6 software. Unpaired ‘t’-tests were used to analyze treatment differences between control groups (sham-surgery and UUO+positive-control 1D11 each *vs*. UUO control). One-way analysis of variance with post-hoc Dunnett’s test was used to compare treatment differences between CVC groups and UUO controls.

## Results

### Effect of CVC on recruitment/migration of monocytes/macrophages

#### *In vivo* mouse model of peritonitis

**Fig 1:**
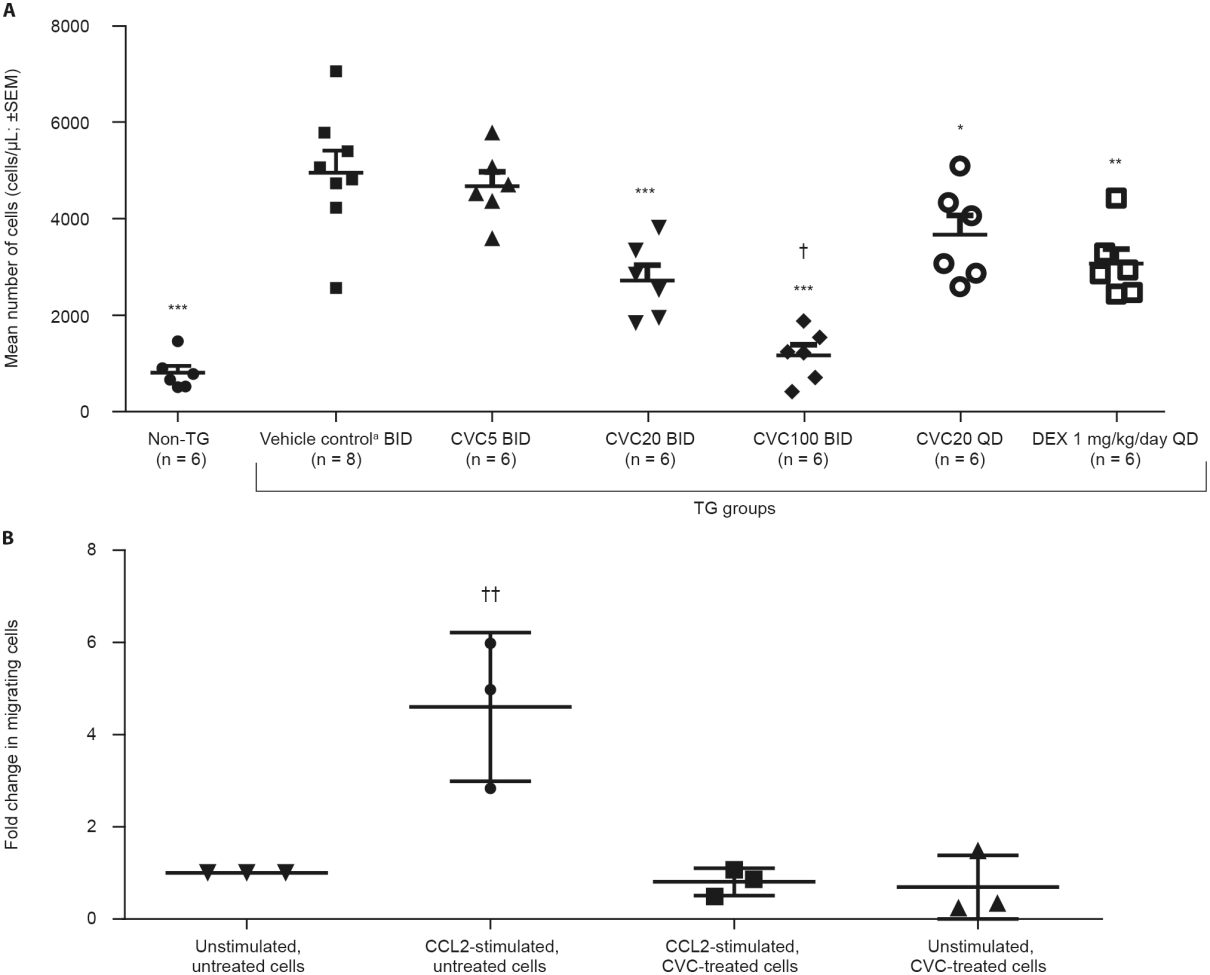
CVC Effect on Monocyte/Macrophage Migration. (A) Monocyte/macrophage recruitment reduced in TG-induced mouse model of peritonitis after pre-treatment with CVC (PO BID or QD dosing); (B) cenicriviroc (1 μM) inhibits CCL2-mediated chemotaxis of activated murine macrophages (F4/80^+^/CD11b^+^) *ex vivo*. *p < 0.05 *vs*. TG + vehicle control; **p < 0.01 *vs*. TG + vehicle control; ***p < 0.001 *vs*. TG + vehicle control; † *p* < 0.001 *vs*. TG + DEX; ‡ *p* = 0.018 *vs*. vehicle control. ^a^Vehicle control: 0.5% [w/v] methylcellulose + 1% Tween^®^-80 (pH ~1.3). BID, twice daily; CCL2, C-C chemokine ligand 2; CVC, cenicriviroc; CVC5, CVC 5 mg/kg/day; CVC20, CVC 20 mg/kg/day; CVC100, CVC 100 mg/kg/day; DEX, dexamethasone; PO, oral gavage; QD, once daily; SEM, standard error of the mean; TG, thioglycollate.

In the TG-induced model of peritonitis, CVC treatment led to dose-related decreases in monocyte/macrophage recruitment, of similar or greater magnitude than those observed with dexamethasone (positive control), and achieving statistical significance at doses ≥20 mg/kg/day (*p* < 0.05; Fig 1A; S1 Fig). Compared to the vehicle-control group, peritoneal lavage monocyte/macrophage counts were decreased by: 5.7%, 45.2%, 76.5%, 26.0% and 38.1% for CVC5 BID, CVC20 BID, CVC100 BID, CVC20 QD and dexamethasone, respectively. Exposure to CVC was dose-related and correlated with the decrease in monocyte/macrophage recruitment, with CVC appearing to be more effective when given BID *versus* QD, in line with the higher plasma concentrations achieved with BID dosing and the known short half-life in mice (~2 hours). Compared to dexamethasone, monocyte/macrophage-count decreases were significantly more pronounced with CVC100 BID (62.1% greater reduction, *p* < 0.001).

#### *Ex vivo* migration of mouse monocytes

Migration of mouse monocytes in response to CCL2, the most potent mediator of chemotaxis for activated macrophages, was reduced following pre-treatment with CVC at a concentration of 1 μM (Fig 1B). Compared to untreated and unstimulated cells, the average fold change in migrating cells (±SD) was 4.6±0.9 (*p* < 0.05), 0.8±0.2 (*p* > 0.05) and 0.7±0.4 (*p* > 0.05) for CCL2-stimulated cells, CCL2-stimulated cells treated with CVC and unstimulated cells treated with CVC, respectively.

### Antifibrotic effects of CVC in animal models of fibrosis

#### CVC effect on body weight and liver or kidney weight

Overall, no notable effects on body weight and liver or kidney weight were observed following CVC administration in animal models of liver and kidney fibrosis (S2 Table). A slight decrease in body weight was observed in the UUO model (5%, CVC20 *vs*. UUO control on Day 5, *p* < 0.05), and in the liver-to-body weight ratio in the TAA model (established fibrosis, CVC30 *vs*. vehicle control) (S2 Table).

#### CVC effect on liver function

**Fig 2:**
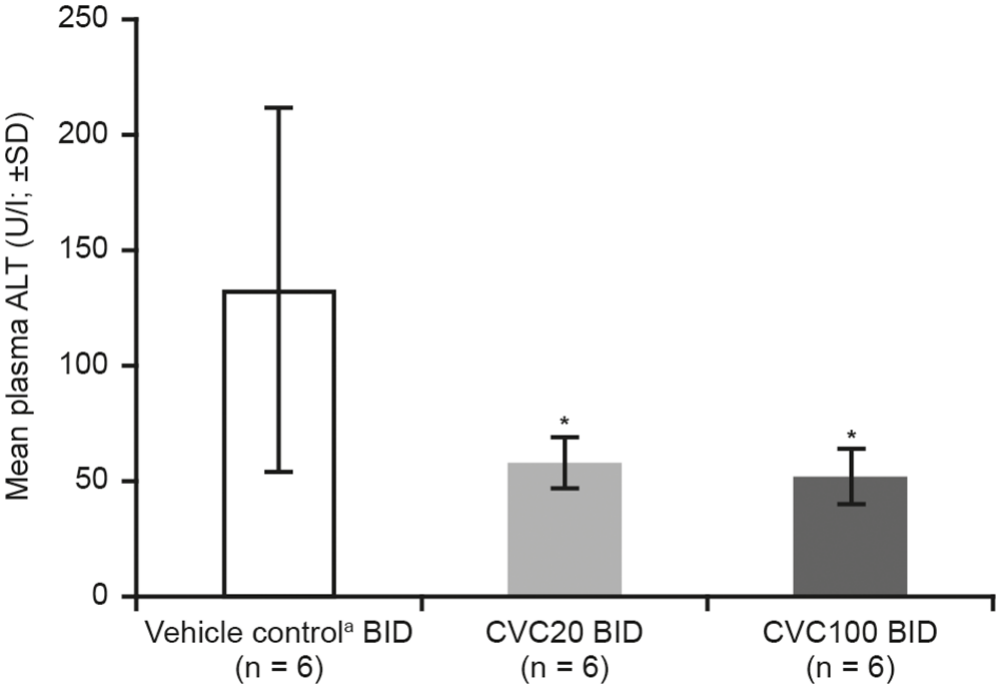
Reduction of Plasma ALT Levels in NASH Model. *p < 0.05 *vs*. vehicle control. ^a^Vehicle control: 0.5% [w/v] methylcellulose + 1% Tween^®^-80 (pH ~1.3). ALT, alanine aminotransferase; BID, twice daily; CVC, cenicriviroc; CVC20, CVC 20 mg/kg/day; CVC100, CVC 100 mg/kg/day; NASH, non-alcoholic steatohepatitis; SD, standard deviation.

In the NASH model, plasma ALT levels were significantly decreased with both CVC doses *versus* vehicle control (*p* < 0.05; Fig 2). AST levels were not assessed.

In the TAA model, no significant differences in ALT levels were observed between the CVC-treated and vehicle-control groups, whereas AST levels were significantly decreased for CVC30 *versus* vehicle control (*p* < 0.05) in the early intervention group (S2 Fig).

#### CVC effect on plasma CCL2 levels

No significant differences in CCL2 levels measured in the NASH model were observed between the vehicle and CVC20 groups, while a significant increase in plasma CCL2 levels was observed in the CVC100 group compared with the vehicle group (vehicle, 60±4 pg/mL; CVC20, 68±16 pg/mL; CVC100, 91±14 pg/mL).

#### CVC effect on morphology of liver/obstructed kidney

**Fig 3:**
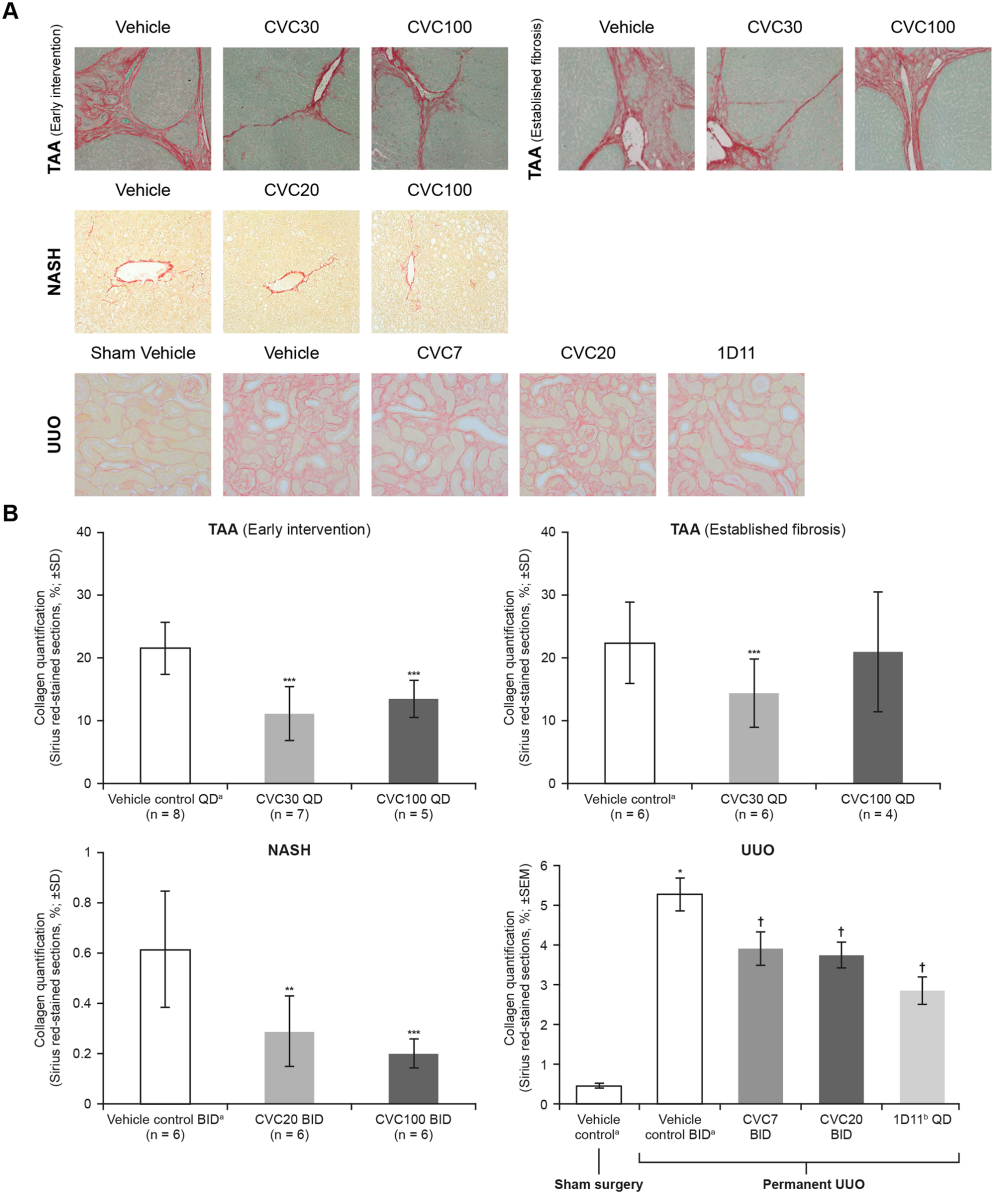
Reduction in Fibrosis. (A) Representative micrographs of Sirius red-stained liver sections in the rat TAA model (early intervention and established fibrosis; 100x), mouse NASH model (200x) and mouse UUO model (200x). (B) Reduction in collagen deposition in the rat TAA model (early intervention and established fibrosis), mouse NASH model^a^ and mouse UUO model^b^. *p < 0.05 *vs*. sham control; **p < 0.01 *vs*. vehicle control; ***p < 0.001 *vs*. vehicle control; †*p* < 0.05 *vs*. UUO + vehicle control. ^a^Perivascular area subtracted; ^b^Data presented exclude a single outlier from an animal in the CVC20 group, which had a CVF value >2 SDs higher than any other animal in the group. CVF, Collagen Volume Fraction; CVC, cenicriviroc; NASH, non-alcoholic steatohepatitis; SD, standard deviation; SEM, standard error of the mean; TAA, thioacetamide; UUO, unilateral ureteral obstruction.

Liver collagen deposition was reduced following CVC treatment started concurrently with TAA, *versus* that following vehicle control (48.5% and 37.5% reduction for CVC30 and CVC100, respectively, *p* < 0.001; Fig 3). When CVC treatment was initiated at the intermediate stage of liver disease (4 weeks after TAA), a significant antifibrotic effect was observed with CVC30 (35.7% reduction *vs*. vehicle control, *p* < 0.001), but not CVC100 (Fig 3B). No significant reduction in collagen deposition was observed in the cirrhosis-reversal group when CVC treatment started at Week 8 (S3 Fig). No histologic differences were observed between groups based on examination of H&E-stained liver sections (S4 Fig).

In the NASH model, collagen deposition in the pericentral region of the liver lobule was markedly reduced in both CVC groups *versus* vehicle control. Liver sections from the vehicle group exhibited severe micro- and macro-vesicular fat deposition, hepatocellular ballooning and inflammatory cell infiltration (S4 Fig). The CVC20 and CVC100 groups displayed moderate improvements in lobular inflammation and hepatocyte ballooning (S4 Fig), with a significant reduction in NAS *versus* that in the vehicle group (*p* < 0.05 and *p* < 0.01, respectively; Table 1). The percentage of fibrosis area was significantly decreased by CVC treatment *versus* vehicle control (40.0% and 41.8% reduction for CVC20 and CVC100, respectively, *p* < 0.01). The modified fibrosis areas (perivascular space subtracted) were also significantly reduced in CVC groups *versus* those in vehicle controls (52.5% and 67.2% reduction for CVC20 and CVC100, respectively, *p* < 0.01; Fig 3).

**Table 1:** Reduction of NAS in NASH Model^a^.

Score	Vehicle control^b^ (n = 6)	CVC20 (n = 6)	CVC100 (n = 6)
Steatosis			
0	0	0	1
1	4	6	5
2	2	0	0
3	0	0	0
Lobular inflammation			
0	0	0	0
1	0	3	3
2	6	3	3
3	0	0	0
Hepatocyte ballooning			
0	0	0	1
1	0	3	2
2	6	3	3
NAS, mean (±SD)	5.3 (±0.5)	4.0 (±0.6)*	3.7 (±0.8)**

*p = 0.0013 *vs*. vehicle control;

**p = 0.0009 *vs*. vehicle control.

^a^Interpretation performed by pathologist blinded by treatment group;

^b^Vehicle control: 0.5% [w/v] methylcellulose + 1% Tween^®^-80 (pH ~1.3).

CVC, cenicriviroc; CVC20, CVC 20 mg/kg/day; CVC100, CVC 100 mg/kg/day; n, number of animals; NAS, non-alcoholic fatty liver disease activity score; NASH, non-alcoholic steatohepatitis; SD, standard deviation.

Of note, F4/80 immunostaining of liver sections form the vehicle group showed accumulation of F4/80+ cells in the liver lobule, but there were no significant differences in the number and size of F4/80+ cells between the vehicle and either CVC20 or CVC100 group, nor in the percentage of inflammation area (F4/80+ area; vehicle, 4.99±1.10%; CVC20, 4.77±1.02%; CVC-high, 4.96±0.60%; S5 Fig). Additional staining of F4/80+ macrophages with CD16/32 (M1 marker) or CD206 (M2 marker) showed no significant difference in the M1/M2 ratio between the vehicle and the CVC treatment groups (vehicle, 99.6±20.2%; CVC20, 112.3±17.0%; CVC100 125.1±21.9%).

In the UUO model, renal cortical fibrosis, expressed as Collagen Volume Fraction (±SEM), was significantly increased in UUO-obstructed kidneys relative to sham control (11.4-fold±1.0, *p* < 0.05). 1D11 (positive control) significantly attenuated these UUO-induced increases (50.3%±7.3 reduction *vs*. UUO control, *p* < 0.05). Likewise, CVC7 and CVC20 significantly attenuated UUO-induced increases when a single outlier (value >2 SD; 1 animal in CVC20 group) was removed (28.6%±8.8 and 31.8%±6.8 reduction *vs*. UUO control, respectively, *p* < 0.05; Fig 3).

#### CVC effect on extracellular matrix protein content

**Fig 4:**
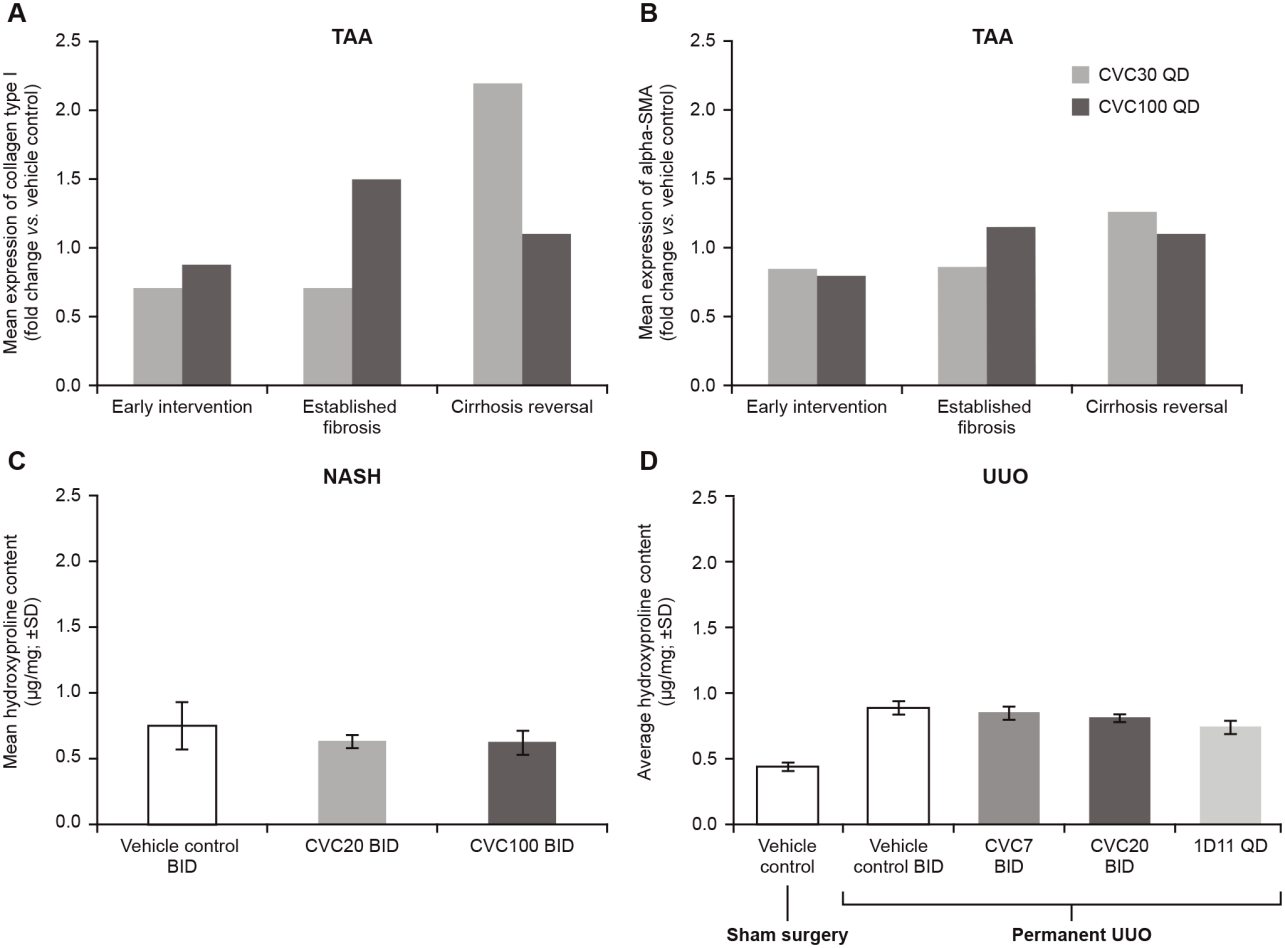
CVC Effects on Extracellular Matrix Protein Content. Mean expression of (A) collagen type I and (B) alpha-SMA in the TAA model compared to vehicle control; mean expression of hydroxyproline content in (C) NASH model and (D) UUO model. alpha-SMA, alpha-smooth muscle actin; BID, twice daily; CVC, cenicriviroc; NASH, non-alcoholic steatohepatitis; QD, once daily; SD, standard deviation; TAA, thioacetamide; UUO, unilateral ureter obstruction.

Compared to vehicle control, protein expression of collagen type 1 in the TAA model was reduced by 29% and 12% for CVC30 and CVC100, respectively, in early intervention, and 30% for CVC30 in established fibrosis. Protein expression of alpha-SMA was reduced following CVC treatment by 17% and 22% for CVC30 and CVC100, respectively, *versus* vehicle control in early intervention, and 14% for CVC30 in established fibrosis. No reductions were observed in protein expression in the advanced disease cirrhosis group (Fig 4). The differences in collagen type 1 and alpha-SMA protein levels between treatment arms were not statistically significant; however, these results likely reflect the significant decrease of fibrosis observed in histological analyses.

In the NASH model, the liver hydroxyproline content tended to decrease in the CVC20 and CVC100 groups compared with the vehicle group (vehicle, 0.75±0.18 μg/mg; CVC20, 0.63±0.05 μg/mg; CVC100, 0.62±0.09 μg/mg; Fig 4).

Compared with sham control, UUO increased hydroxyproline content in obstructed kidneys (1.6±0.2 fold; vehicle control). 1D11 attenuated these increases (37.1±4.9%); however, CVC did not affect these increases in obstructed kidney hydroxyproline content (Fig 4).

#### CVC effect on gene expression of fibrotic/inflammatory biomarkers

In the TAA model, there were no significant changes between CVC and vehicle-control groups in mRNA expression of the fibrosis markers assessed (S6 Fig).

Collagen type 1 mRNA expression levels in the NASH model decreased with CVC treatment (37% and 27% reduction *vs*. vehicle control for CVC20 and CVC100, respectively). This reduction was statistically significant only for the CVC20 group (*p* < 0.05). There were no statistically significant differences in mRNA expression levels between the vehicle-control and CVC groups for the other fibrosis markers tested in this model (S7 Fig).

In the UUO model, UUO significantly increased collagen 1a1, collagen 3a1, fibronectin-1 and TGF-beta_1_ mRNA expression in vehicle-treated animals *versus* sham controls, and 1D11 (positive control) significantly attenuated these UUO-induced increases (*p* < 0.05) (S8 Fig). There was a trend for CVC7 and CVC20 to inhibit UUO-induced increases in the obstructed renal cortical mRNA expression of some fibrogenic genes; however, it did not reach statistical significance relative to UUO control.

### CVC PK analysis

In the TG-induced model of peritonitis, following BID dosing, plasma levels of CVC ~14 hours post-dose (trough levels) increased in a dose-dependent manner (Table 2). At CVC20, lower trough plasma levels were seen with QD (n = 5) *versus* BID dosing (n = 5) (25.7±8.1 ng/mL [26 hours post-dose] *vs*. 81.2±22.8 ng/mL [14 hours post-dose], respectively).

**Table 2:** CVC Plasma Trough Levels (Mean±SD).

Dose	Time post-dose	CVC concentration (mean±SD, ng/mL)
**TG-induced mouse model of peritonitis (Day 6)**		
CVC5 BID, n = 2	~14 hours	13.5^a^
CVC20 BID, n = 5	~14 hours	81.2±22.8
CVC100 BID, n = 5	~14 hours	551±253
**Mouse model of UUO-induced renal fibrosis (Day 5)**		
CVC7 BID, n = 8	~4 hours	251±364
CVC20 BID, n = 8	~4 hours	971±507

^a^3 of 5 samples below the lower limit of quantification (10 ng/mL).

BID, twice-daily; CVC, cenicriviroc; CVC5, CVC 5 mg/kg/day; CVC7, CVC 7 mg/kg/day; CVC20, CVC 20 mg/kg/day; CVC100, CVC 100 mg/kg/day; n, number of animals; SD, standard deviation; TG, thioglycollate; UUO, unilateral ureter obstruction

In the UUO model, all dose groups had exposure to CVC and plasma levels of CVC at the approximate maximum concentration (C_max_) 4 hours post-dose on Day 5, and these increased in a dose-dependent manner (Table 2).

## Discussion

CVC displayed significant anti-inflammatory and antifibrotic effects across a range of *in vivo* models, including liver fibrosis, NASH and kidney fibrosis. CVC reduced monocyte/macrophage recruitment in an *in vivo* model of peritonitis, and monocyte migration *in vitro*. A significant decrease in monocyte/macrophage recruitment *versus* vehicle control (*p* < 0.05) and the positive control dexamethasone (*p* < 0.001) was obtained following treatment with CVC100 BID *in vivo*. These findings support CVC’s anti-inflammatory mode of action, and are further substantiated by results from the NASH model, where a significantly lower NAS score was observed in the CVC treatment groups relative to the vehicle-control group and a greater number of CVC-treated animals had decreased lobular inflammation and prominent hepatocellular ballooning.

In all models, reductions in collagen deposition and production were observed (a significant reduction in collagen deposition, as well as decreased protein and mRNA expression of collagen type 1). While the significant decreases in liver collagen deposition were dose-dependent in the NASH model, where CVC was administered BID given its short half-life in mice (~2 hours), CVC30 in the TAA model lead to the greater reduction in collagen deposition *versus* CVC100 (48.5% and 37.5% reduction *vs*. vehicle control, respectively, *p* < 0.001). The QD administration of CVC in the TAA model and short half-life of CVC in rats (4–5 hours) may have led to increased clearance of CVC, explaining the lack of apparent dose response. In the UUO model, where gavage was performed twice daily in mice, a significant antifibrotic effect was observed at the low dose (CVC7; *p* < 0.05) suggesting that this could represent the minimum effective dose. The lack of apparent dose response in this model may also be explained by the short treatment duration (5 days) and the fact that adequate and sustained receptor occupancy (necessary to disrupt CCL2- and CCL5-induced migration) may have already been achieved at lower CVC doses. In fact, CCR2 and CCR5 receptor occupancy by CVC was assessed *ex vivo* on murine cryopreserved peripheral blood mononuclear cells by Lishomwa Ndlhovu and colleagues [39], in which a CVC concentration of 250 nM achieved >90% and 87% occupancy for CCR2 (blood and spleen) and CCR5 (spleen), respectively, in proinflammatory monocytes.

CCR2 and CCR5 have become attractive targets for antifibrotic therapy, as interactions with their ligands, including CCL2 and CCL5, mediate recruitment of inflammatory cells to the site of liver injury, and contribute to fibrosis [9–15,40]. CVC has previously been shown to initiate high CCR2/CCR5 occupancy on mouse and human monocytes [28,29], and to lead to reciprocal increases in CCL2 in both species [25,26]. In this study, an increase in CCL2 was similarly observed in the CVC100 group compared with the vehicle-control group in the NASH model. The findings reported here show that CVC dual CCR2/CCR5 antagonism causes a reduction in the recruitment and migration of pro-inflammatory monocytes/macrophages, ultimately resulting in decreased fibrogenesis, as measured by collagen deposition and gene/protein expression of collagen type 1. In line with this, CVC does not appear to impact fibrolysis, as shown in the TAA model, where reductions in collagen deposition, as well as in collagen type 1 and alpha-SMA protein expression, were observed in the early intervention and established fibrosis groups, but not the cirrhosis-reversal group. These data suggest that CVC may be best suited for preventing fibrosis progression or improving fibrosis regression, rather than reversing well-established cirrhosis.

CVC was also well tolerated in disease models and had no deleterious effects on body and liver/kidney weight, and liver function. Notably, significant decreases in plasma ALT (NASH model) and AST (early intervention in TAA model) occurred, indicating potential reduction in liver damage with CVC.

One of the limitations of this paper is that, while evaluation of CVC in these animal models demonstrated significant antifibrotic activity, the underlying mechanisms that led to these findings (e.g. effects on monocyte/macrophage infiltration, specific monocyte/macrophage subsets and phenotypes in the liver or kidney, effects on Kupffer cells, HSCs, pericytes or fibroblasts) were not fully elucidated. For example, macrophage staining was only performed in the NASH model, and flow cytometry was not conducted on liver tissue to evaluate the effects of CVC on the infiltrating inflammatory monocyte subset characterized by high expression of CCR2, specifically Ly6C^hi^. Another limitation is the fact that the experiments presented here were conducted by various groups, using different methodologies and on models that are not widely used, which may limit interpretation of findings.

The effects of CVC are currently being evaluated in established animal models of acute and chronic hepatic injury, such as the acetaminophen (APAP), carbon tetrachloride (CCl4) and methionine and choline deficient diet (MCD)-induced models [41–43]. Preliminary data have demonstrated that CVC treatment significantly decreased infiltration of Ly6C^hi^ monocyte-derived macrophages into the liver in all three models [42]. CVC treatment was also associated with a significant protection from acute APAP-induced liver injury, with a significant reduction in ALT levels and in necrotic area relative to vehicle control. A significant reduction in necrotic area was also observed in the acute CCl4 model. In the chronic MCD model of NASH, CVC treatment significantly ameliorated steatohepatitis, as assessed by the histological NAFLD activity score, and reduced hepatic fibrosis, as evidenced by decreased Sirius red staining and hydroxyproline content.

CVC has completed Phase 2b clinical development for treatment of HIV-1 infection in antiretroviral treatment-naïve adults with CCR5-tropic virus (Study 652-2-202; NCT01338883) [26]. CVC provided potent CCR2/CCR5 blockade and showed antifibrotic properties. Indeed, the proportion of subjects with aspartate aminotransferase-to-platelet count ratio index (APRI) score ≥0.5 and non-invasive hepatic fibrosis risk (FIB-4) score ≥1.45 decreased by 75% and 73%, respectively, between Baseline and Week 24; these decreases were maintained at Week 48 [44]. More recently, an independent analysis of study samples showed that CVC treatment led to significant reduction in the enhanced liver fibrosis (ELF) scores at Week 48 (*p* < 0.0001) [45]. In addition, CVC exhibited a good tolerability profile in humans, including hepatically impaired subjects [21,25,26]. Overall, these results establish proof of principle for human testing of CVC for fibrosis-related conditions, including NASH.

NASH, a hepatic manifestation of the metabolic syndrome typically associated with insulin resistance, is the most serious form of highly prevalent NAFLD [46–48]. Mild to moderate fibrosis is present in 76–100% of patients with NASH, and severe fibrosis in 15–50%; cirrhosis affects 7–16% of adults with the condition [49]. CVC 150 mg QD is in Phase 2b clinical development for the treatment of adults with NASH and liver fibrosis (CENTAUR Study 652-2-203; NCT02217475). No drugs are currently approved to treat NASH but, in addition to CVC, several other agents are under investigation. These have different mechanisms of action to CVC, and include high-dose vitamin E, pioglitazone (peroxisome proliferator-activated receptor [PPAR]-gamma agonist), obeticholic acid (a farnesoid X receptor agonist) and elafibranor (formerly GFT505, a peroxisome proliferator-activated receptor-alpha/delta agonist) [50–52]. Pioglitazone, obeticholic acid and elafibranor have shown amelioration of insulin resistance and/or antifibrotic activity in animal models [53–55] and have undergone Phase 2 clinical evaluation [50,51,56] (NCT01694849). Taken together, these findings provide a strong rationale for targeting both inflammatory and metabolic pathways in NASH, and warrant the need to evaluate combination therapies as a means to further improve treatment outcomes.

In conclusion, the comprehensive and consistent preclinical data demonstrating anti-inflammatory and antifibrotic effects of CVC, existing human safety data, oral availability, and therapeutic targets strongly implicated in experimental and human liver diseases [9–15] provide a strong rationale to further evaluate CVC as a treatment for NASH with liver fibrosis.

## Supporting Information

S1 Table Experimental Designs. ^a^The number of animals at the start of the study is indicated, with the number of animals at the end of the study on which analyses were conducted indicated in brackets; ^b^Vehicle control: 0.5% [w/v] methylcellulose + 1% Tween^®^-80. BID, twice daily; CVC, cenicriviroc; DEX, dexamethasone; IP, intraperitoneal; NASH, non-alcoholic steatohepatitis; PBS, phosphate buffer saline; PO, oral gavage; QD, once daily; SC, subcutaneous; STAM, stelic animal model; TAA, thioacetamide; TG, thioglycollate; UUO, unilateral ureter obstruction.
(DOCX)

S2 Table Effects of CVC on Body, Liver or Kidney Weight. *p < 0.05 vs. vehicle control; CVC, cenicriviroc; DEX, dexamethasone; NASH, non-alcoholic steatohepatitis; SD, standard deviation; SEM; standard error of mean; TAA, thioacetamide; UUO, unilateral ureter obstruction.
(DOCX)

S1 Fig Representative Plots of Peritonitis Lavage Cell Counts. Total and differential cell counts were assessed in peritoneal lavage samples using an Advia^®^ Hematology System (Siemens Healthcare Diagnostics, USA) with multispecies software and an analysis software designed for mouse peritoneal fluid on Advia^®^ 120 (LabThruPut, New York, USA). The software applies cluster analysis on the two channels (peroxidase and basophil channels) pictured. In the peroxidase channel, eosinophils are shown in yellow, neutrophils in magenta and mononuclear cells (lymphocytes, monocytes and macrophages) in cyan. In the basophil channel, neutrophils and eosinophils are shown in magenta and cellular debris in white. Information from both channels are combined to obtain mononuclear cells and neutrophil counts. The peritoneal fluid white-blood-cell count, and the absolute and differential mononuclear cell, neutrophil and eosinophil counts are then calculated. BID, twice daily; CVC, cenicriviroc; CVC5, CVC 5 mg/kg/day; CVC20, CVC 20 mg/kg/day; CVC100, CVC 100 mg/kg/day; QD, once daily; TG, thioglycollate.
(TIF)

S2 Fig CVC effects on Liver Function in the TAA Model. (A) Average ALT levels and (B) Average AST levels in the early intervention, established fibrosis and cirrhosis reversal groups. *p < 0.05 *vs*. vehicle control; ALT, alanine aminotransferase; AST, aspartate aminotransferase; CVC, cenicriviroc; QD, once daily; SD, standard deviation; TAA, thioacetamide.
(TIF)

S3 Fig Liver Collagen Deposition in the Cirrhosis Reversal Intervention (TAA Model). CVC, cenicriviroc; QD, once daily; SD, standard deviation; TAA, thioacetamide.
(TIF)

S4 Fig Histological Examination of H&E Stained Liver Sections. Representative micrographs of H&E-stained liver sections in (A) the rat TAA model (40x) and (B) the mouse NASH model (50x). CVC, cenicriviroc; H&E, hematoxylin and eosin; NASH, non-alcoholic steatohepatitis; TAA, thioacetamide.
(TIF)

S5 Fig Representative F4/80 micrographs in the NASH model. CVC, cenicriviroc; NASH, non-alcoholic steatohepatitis.
(TIF)

S6 Fig mRNA Expression of Fibrosis Markers in the TAA Model. (A) Collagen type I; (B) alpha-SMA; (C) beta-PDGFR; (D) TGF-beta; (E) MMP2; (F) TIMP1; (G) TIMP2. CVC, cenicriviroc; MMP2, matrix metalloproteinase 2; beta-PDGFR, beta-platelet-derived growth factor-beta receptor; QD, once daily; SE, standard error; alpha-SMA, alpha-smooth muscle actin; TAA, thioacetamide; TGF-beta, transforming growth factor-beta; TIMP, tissue inhibitor of metalloproteinase.
(TIF)

S7 Fig mRNA Expression of Fibrosis Markers in the NASH Model. *p < 0.05 *vs*. vehicle control; BID, twice daily; CVC, cenicriviroc; MCP-1, monocyte chemotactic protein-1; NASH, non-alcoholic steatohepatitis; SD, standard deviation; TIMP, tissue inhibitor of metalloproteinase; TNF, tumor necrosis factor.
(TIF)

S8 Fig mRNA Expression of Fibrosis Markers in the UUO Model. (A) Collagen 1a1; (B) Collagen 3a1; (C) alpha-SMA; (D) TGF-beta; (E) MCP-1; (F) Fibronectin; (G) CTFG. *p < 0.05 *vs*. sham control; ^†^*p* < 0.05 *vs*. UUO control; BID, twice daily; CTFG, connective tissue growth factor; CVC, cenicriviroc; MCP-1, monocyte chemotactic protein-1; QD, once daily; SEM, standard error of the mean; alpha-SMA, alpha-smooth muscle actin; TGF-beta, transforming growth factor-beta; UUO, unilateral ureter obstruction.
(TIF)
