# Supplementary material for: Antifibrotic Effects of the Dual CCR2/CCR5 Antagonist Cenicriviroc in Animal Models of Liver and Kidney Fibrosis
Source: PLoS One. 2016 Jun 27;11(6):e0158156. doi: 10.1371/journal.pone.0158156 (PMC4922569; doi:10.1371/journal.pone.0158156)
Supplement: S1 Table — aThe number of animals at the start of the study is indicated, with the number of animals at the end of the study on which analyses were conducted indicated in brackets; bVehicle control: 0.5% [w/v] methylcellulose + 1% Tween®-80. BID, twice daily; CVC, cenicriviroc; DEX, dexamethasone; IP, intraperitoneal; NASH, non-alcoholic steatohepatitis; PBS, phosphate buffer saline; PO, oral gavage; QD, once daily; SC, subcutaneous; STAM, stelic animal model; TAA, thioacetamide; TG, thioglycollate; UUO, unilateral ureter obstruction. (DOCX) [file pone.0158156.s001.docx]

S1 Table. Experimental designs.

| Group | Treatment regimen | Sacrifice | N (n_final_)^a^ |
| --- | --- | --- | --- |
| **Effect of CVC in the TG-induced peritonitis model in mice** | | | |
| Non-TG + vehicle control | Days 1–5: vehicle control^b^ PO  Day 4: PBS IP | Day 6 | 6 (6) |
| TG + vehicle control | Days 1–5: vehicle control^b^ BID PO  Day 4: 3.85% TG 1 mL IP | Day 6 | 8 (8) |
| TG + CVC 5 mg/kg/day | Days 1–5: CVC 2.5 mg/kg/dose BID PO  Day 4: 3.85% TG 1 mL IP | Day 6 | 6 (6) |
| TG + CVC 20 mg/kg/day | Days 1–5: CVC 10 mg/kg/dose BID PO  Day 4: 3.85% TG 1 mL IP | Day 6 | 6 (6) |
| TG + CVC 100 mg/kg/day | Days 1–5: CVC 50 mg/kg/dose BID PO  Day 4: 3.85% TG 1 mL IP | Day 6 | 6 (6) |
| TG + CVC 20 mg/kg/day | Days 1–5: CVC 20 mg/kg/dose QD PO  Day 4: 3.85% TG 1 mL IP | Day 6 | 6 (6) |
| TG + DEX 1 mg/kg/day | Days 1–5: DEX 1 mg/kg/dose QD PO  Day 4: 3.85% TG 1 mL IP | Day 6 | 6 (6) |
| **Activity of CVC in a rat model of TAA-induced liver fibrosis and cirrhosis** | | | |
| *Group 1: Early intervention* | | | |
| Vehicle control | Weeks 0–8: TAA 150 mg/kg 3 times/week IP Weeks 0–8: vehicle control^b^ QD PO | Week 8 | 8 (8) |
| CVC 30 mg/kg/day | Weeks 0–8: TAA 150 mg/kg 3 times/week IP  Weeks 0–8: CVC 30 mg/kg/dose QD PO | Week 8 | 8 (7) |
| CVC 100 mg/kg/day | Weeks 0–8: TAA 150 mg/kg 3 times/week IP  Weeks 0–8: CVC 100 mg/kg/dose QD PO | Week 8 | 8 (5) |
| *Group 2: Established fibrosis* | | | |
| Vehicle control | Weeks 0–8: TAA 150 mg/kg 3 times/week IP  Weeks 4–8: vehicle control^b^ QD PO | Week 8 | 8 (6) |
| CVC 30 mg/kg/day | Weeks 0–8: TAA 150 mg/kg 3 times/week IP  Weeks 4–8: CVC 30 mg/kg/dose QD PO | Week 8 | 8 (6) |
| CVC 100 mg/kg/day | Weeks 0–8: TAA 150 mg/kg 3 times/week IP  Weeks 4–8: CVC 100 mg/kg/dose QD PO | Week 8 | 8 (4) |
| *Group 3: Cirrhosis reversal* | | | |
| Vehicle control | Weeks 0–8: TAA 150 mg/kg 3 times/week IP  Weeks 8–12: vehicle control^b^ QD PO | Week 12 | 8 (7) |
| CVC 30 mg/kg/day | Weeks 0–8: TAA 150 mg/kg 3 times/week IP  Weeks 8–12: CVC 30 mg/kg/dose QD PO | Week 12 | 8 (7) |
| CVC 100 mg/kg/day | Weeks 0–8: TAA 150 mg/kg 3 times/week IP  Weeks 8–12: CVC 100 mg/kg/dose QD PO | Week 12 | 8 (8) |
| ***In vivo* efficacy study of CVC in STAM model of NASH** | | | |
| Vehicle control | Day 2: streptozotocin 200 µg SC  Weeks 6­–9: vehicle control^b^ BID PO | Week 9 | 9 (6) |
| CVC 20 mg/kg/day | Day 2: streptozotocin 200 µg SC  Weeks 6­–9: CVC 10 mg/kg/dose BID PO | Week 9 | 9 (6) |
| CVC 100 mg/kg/day | Day 2: streptozotocin 200 µg SC  Weeks 6­–9: CVC 50 mg/kg/dose BID PO | Week 9 | 9 (6) |
| **Dose-dependent effects of CVC on renal biomarkers and fibrosis in UUO mice** | | | |
| Vehicle control (Sham surgery) | Day 0: sham surgery  Days 0–5: vehicle control^b^ 10 mL/kg BID PO  Day -1–4: PBS IP | Day 5 | 6 (6) |
| Vehicle control  (Permanent UUO) | Day 0: permanent UUO  Days 0–5: vehicle control^b^ 10 mL/kg BID PO  Day -1–4: PBS IP | Day 5 | 9 (9) |
| CVC 7 mg/kg/day | Day 0: permanent UUO  Days 0–5: CVC 3.5 mg/kg/dose BID PO  Day -1–4: PBS IP | Day 5 | 9 (8) |
| CVC 20 mg/kg/day | Day 0: permanent UUO  Days 0–5: CVC 10 mg/kg/dose BID PO  Day -1–4: PBS IP | Day 5 | 9 (8) |
| CVC 100 mg/kg/day | Day 0: permanent UUO  Days 0–5: CVC 50 mg/kg/dose BID PO  Day -1–4: PBS IP | Day 5 | 9 (0) |
| 1D11 3 mg/kg/day | Day 0: permanent UUO  Days 0–5: vehicle control^b^ 10 mL/kg BID PO  Day -1–4: 1D11 3 mg/kg IP | Day 5 | 9 (9) |

^a^The number of animals at the start of the study is indicated, with the number of animals at the end of the study on which analyses were conducted indicated in brackets; ^b^Vehicle control: 0.5% [w/v] methylcellulose + 1% Tween^®^-80. BID, twice daily; CVC, cenicriviroc; DEX, dexamethasone; IP, intraperitoneal; NASH, non-alcoholic steatohepatitis; PBS, phosphate buffer saline; PO, oral gavage; QD, once daily; SC, subcutaneous; STAM, stelic animal model; TAA, thioacetamide; TG, thioglycollate; UUO, unilateral ureter obstruction.
