# Supplementary material for: Antifibrotic Effects of the Dual CCR2/CCR5 Antagonist Cenicriviroc in Animal Models of Liver and Kidney Fibrosis
Source: PLoS One. 2016 Jun 27;11(6):e0158156. doi: 10.1371/journal.pone.0158156 (PMC4922569; doi:10.1371/journal.pone.0158156)
Supplement: S2 Table — *p < 0.05 vs. vehicle control; CVC, cenicriviroc; DEX, dexamethasone; NASH, non-alcoholic steatohepatitis; SD, standard deviation; SEM; standard error of mean; TAA, thioacetamide; UUO, unilateral ureter obstruction. (DOCX) [file pone.0158156.s002.docx]

S2 Table. Effects of CVC on body, liver or kidney weight.

| TAA model | | | | | | |
| --- | --- | --- | --- | --- | --- | --- |
| **Parameter (average±SD)** | **Final body weight (g)** | | **Liver weight (mg)** | | **Liver-to-body  weight ratio (%)** | |
| *Group 1: Early intervention* | | | | | | |
| Vehicle control | | 347.0±7.5 | | 17.6±0.5 | | 5.1±0.2 |
| CVC 30 mg/kg/day | | 330.9±6.4 | | 17.9±1.6 | | 5.4±0.5 |
| CVC 100 mg/kg/day | | 347.2±6.9 | | 17.5±0.5 | | 5.0±0.1 |
| *Group 2: Established fibrosis* | | | | | | |
| Vehicle control | | 336.3±6.3 | | 16.9±0.4 | | 5.0±0.1 |
| CVC 30 mg/kg/day | | 342.0±7.7 | | 15.6±0.6 | | 4.6±0.1* |
| CVC 100 mg/kg/day | | 327.3±7.8 | | 15.8±0.9 | | 4.8±0.2 |
| *Group 3: Cirrhosis reversal* | | | | | | |
| Vehicle control | | 394.0±7.7 | | 20.0±0.7 | | 5.1±0.2 |
| CVC 30 mg/kg/day | | 390.3±9.3 | | 19.0±0.7 | | 4.9±0.2 |
| CVC 100 mg/kg/day | | 386.4±4.9 | | 18.5±0.7 | | 4.8±0.2 |
| **NASH model** | | | | | | |
| **Parameter  (mean±SD)** | **Final body weight (g)** | | **Liver weight (mg)** | | **Liver-to-body  weight ratio (%)** | |
| Vehicle control | | 18.9±3.3 | | 1270±326 | | 6.6±0.8 |
| CVC 20 mg/kg/day | | 19.5±2.0 | | 1334±99 | | 6.9±1.0 |
| CVC 100 mg/kg/day | | 18.7±0.9 | | 1307±119 | | 7.0±0.8 |
| **UUO model** | | | | | | |
| **Parameter (average±SEM)** | **Final body weight (g)** | | **Obstructed kidney weight (mg)** | | **Contralateral kidney weight (mg)** | |
| Vehicle control (Sham) | | 28.2±0.8 | | 198.2±4.8 | | 187.1±7.6 |
| Vehicle control (UUO) | | 27.1±0.2 | | 214.9±10.8 | | 219.3±6.4 |
| CVC 7 mg/kg/day | | 26.3±0.5 | | 232.0±11.9 | | 221.0±10.1 |
| CVC 20 mg/kg/day | | 25.7±0.4 | | 217.0±6.7 | | 202.4±6.1 |
| 1D11 3 mg/kg/day | | 26.9±0.3 | | 232.4±10.5 | | 217.7±7.0 |

**p* < 0.05 *vs.* vehicle control; CVC, cenicriviroc; DEX, dexamethasone; NASH, non-alcoholic steatohepatitis; SD, standard deviation; SEM; standard error of mean; TAA, thioacetamide; UUO, unilateral ureter obstruction
