# Supplementary figures and images for: Antifibrotic Effects of the Dual CCR2/CCR5 Antagonist Cenicriviroc in Animal Models of Liver and Kidney Fibrosis
Source: PLoS One. 2016 Jun 27;11(6):e0158156. doi: 10.1371/journal.pone.0158156 (PMC4922569; doi:10.1371/journal.pone.0158156)

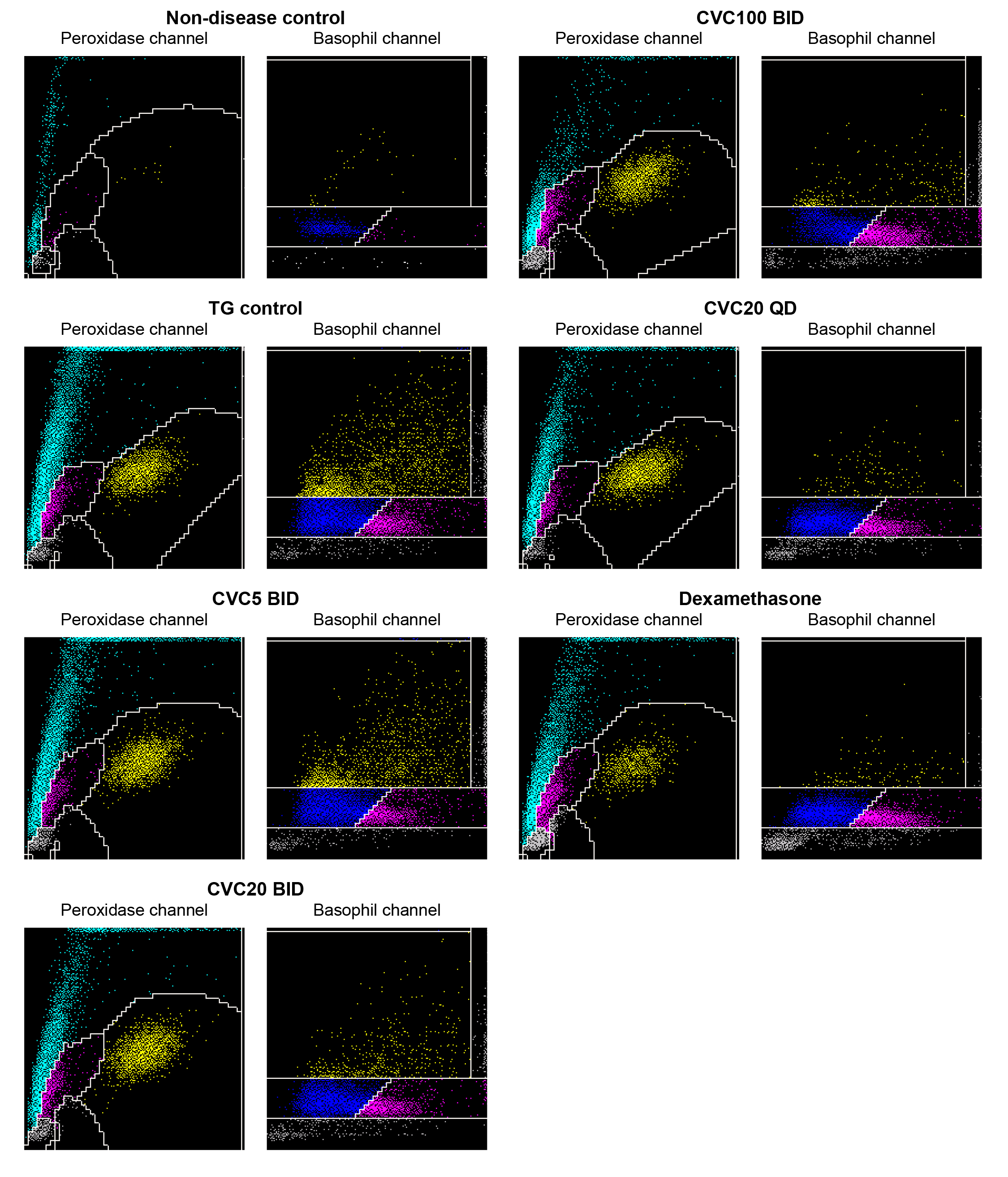

Supplement: S1 Fig — Total and differential cell counts were assessed in peritoneal lavage samples using an Advia® Hematology System (Siemens Healthcare Diagnostics, USA) with multispecies software and an analysis software designed for mouse peritoneal fluid on Advia® 120 (LabThruPut, New York, USA). The software applies cluster analysis on the two channels (peroxidase and basophil channels) pictured. In the peroxidase channel, eosinophils are shown in yellow, neutrophils in magenta and mononuclear cells (lymphocytes, monocytes and macrophages) in cyan. In the basophil channel, neutrophils and eosinophils are shown in magenta and cellular debris in white. Information from both channels are combined to obtain mononuclear cells and neutrophil counts. The peritoneal fluid white-blood-cell count, and the absolute and differential mononuclear cell, neutrophil and eosinophil counts are then calculated. BID, twice daily; CVC, cenicriviroc; CVC5, CVC 5 mg/kg/day; CVC20, CVC 20 mg/kg/day; CVC100, CVC 100 mg/kg/day; QD, once daily; TG, thioglycollate. (TIF) [file pone.0158156.s003.tif]

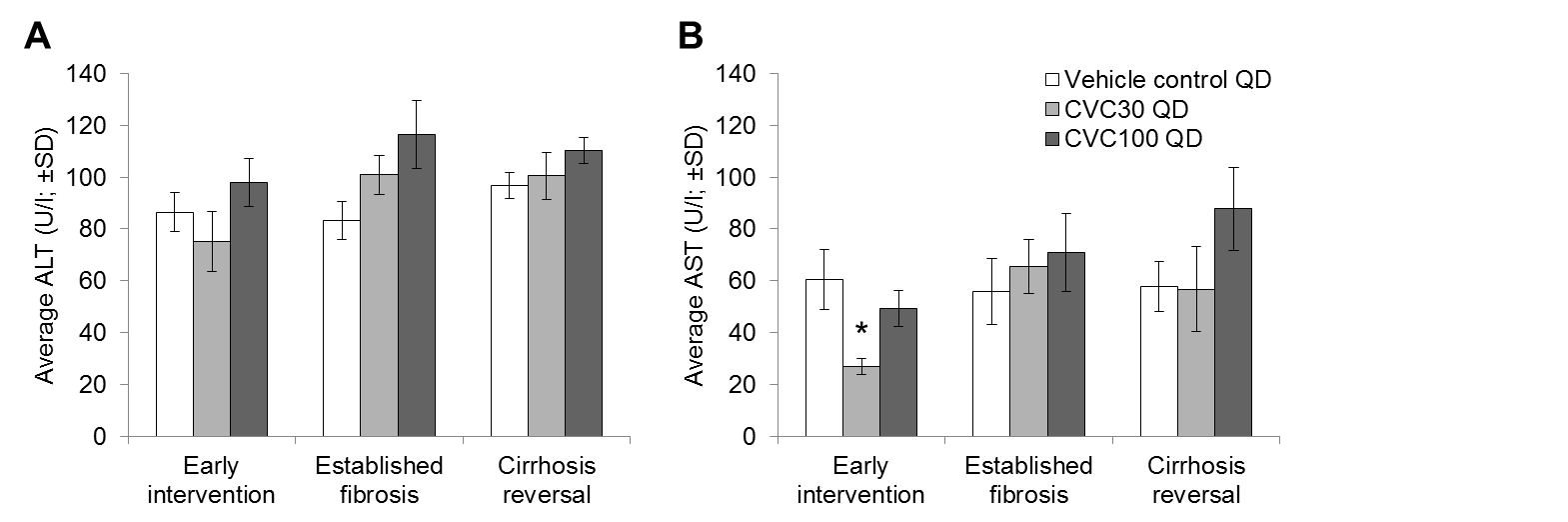

Supplement: S2 Fig — (A) Average ALT levels and (B) Average AST levels in the early intervention, established fibrosis and cirrhosis reversal groups. *p < 0.05 vs. vehicle control; ALT, alanine aminotransferase; AST, aspartate aminotransferase; CVC, cenicriviroc; QD, once daily; SD, standard deviation; TAA, thioacetamide. (TIF) [file pone.0158156.s004.tif]

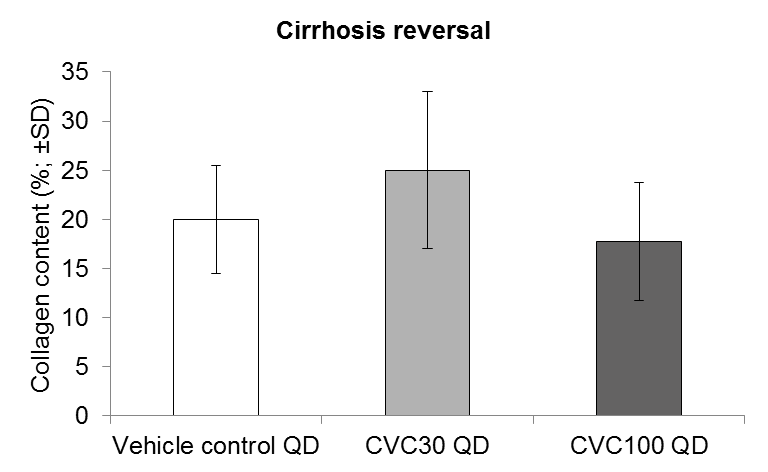

Supplement: S3 Fig — CVC, cenicriviroc; QD, once daily; SD, standard deviation; TAA, thioacetamide. (TIF) [file pone.0158156.s005.tif]

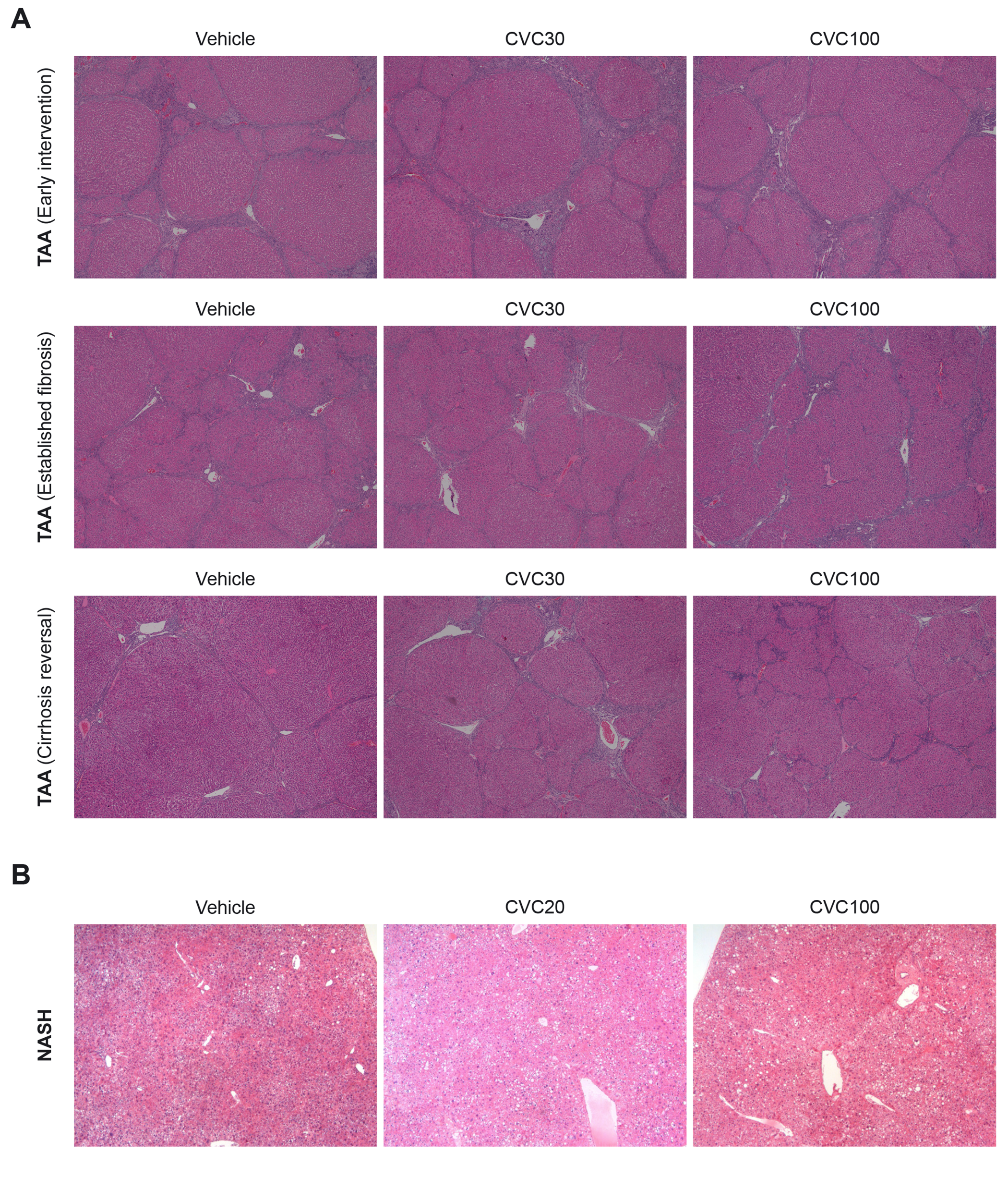

Supplement: S4 Fig — Representative micrographs of H&E-stained liver sections in (A) the rat TAA model (40x) and (B) the mouse NASH model (50x). CVC, cenicriviroc; H&E, hematoxylin and eosin; NASH, non-alcoholic steatohepatitis; TAA, thioacetamide. (TIF) [file pone.0158156.s006.tif]

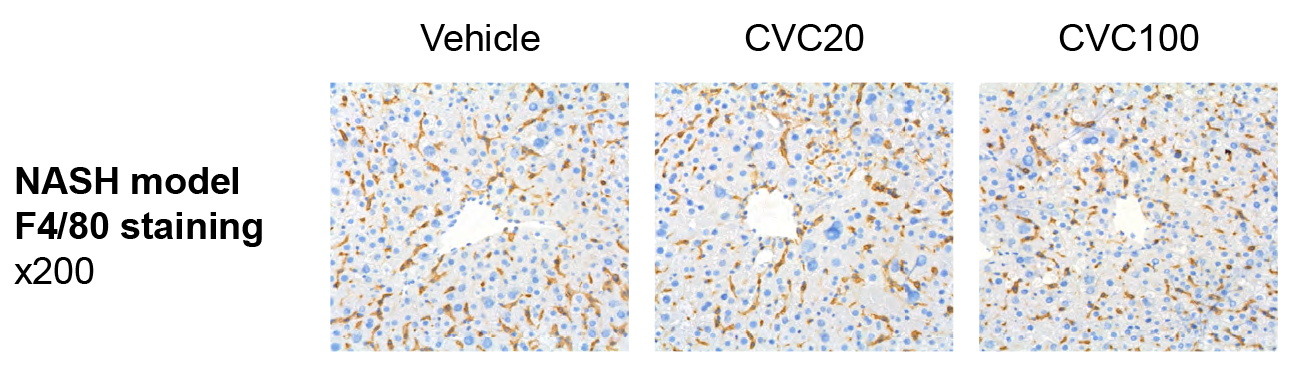

Supplement: S5 Fig — CVC, cenicriviroc; NASH, non-alcoholic steatohepatitis. (TIF) [file pone.0158156.s007.tif]

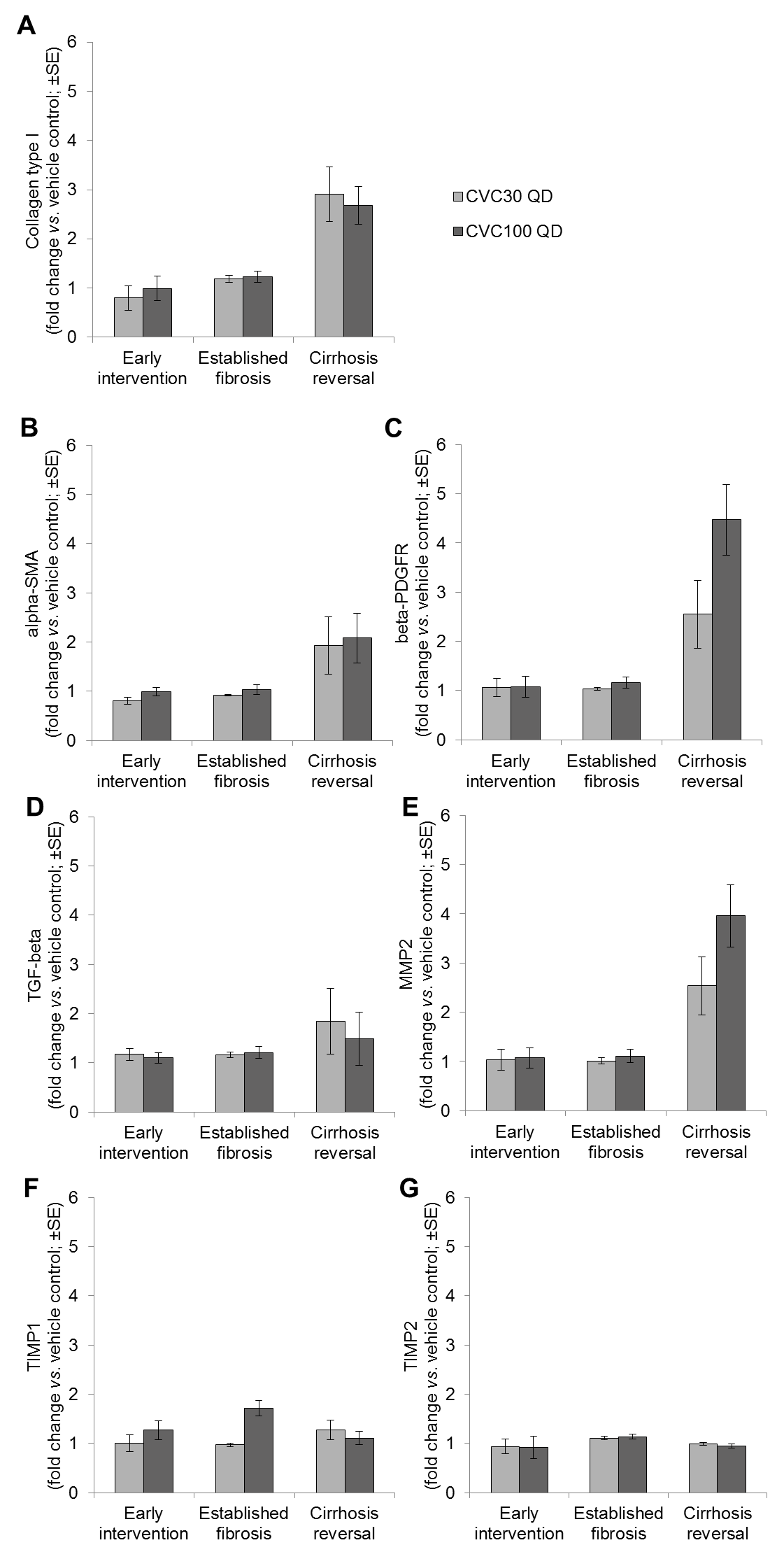

Supplement: S6 Fig — (A) Collagen type I; (B) alpha-SMA; (C) beta-PDGFR; (D) TGF-beta; (E) MMP2; (F) TIMP1; (G) TIMP2. CVC, cenicriviroc; MMP2, matrix metalloproteinase 2; beta-PDGFR, beta-platelet-derived growth factor-beta receptor; QD, once daily; SE, standard error; alpha-SMA, alpha-smooth muscle actin; TAA, thioacetamide; TGF-beta, transforming growth factor-beta; TIMP, tissue inhibitor of metalloproteinase. (TIF) [file pone.0158156.s008.tif]

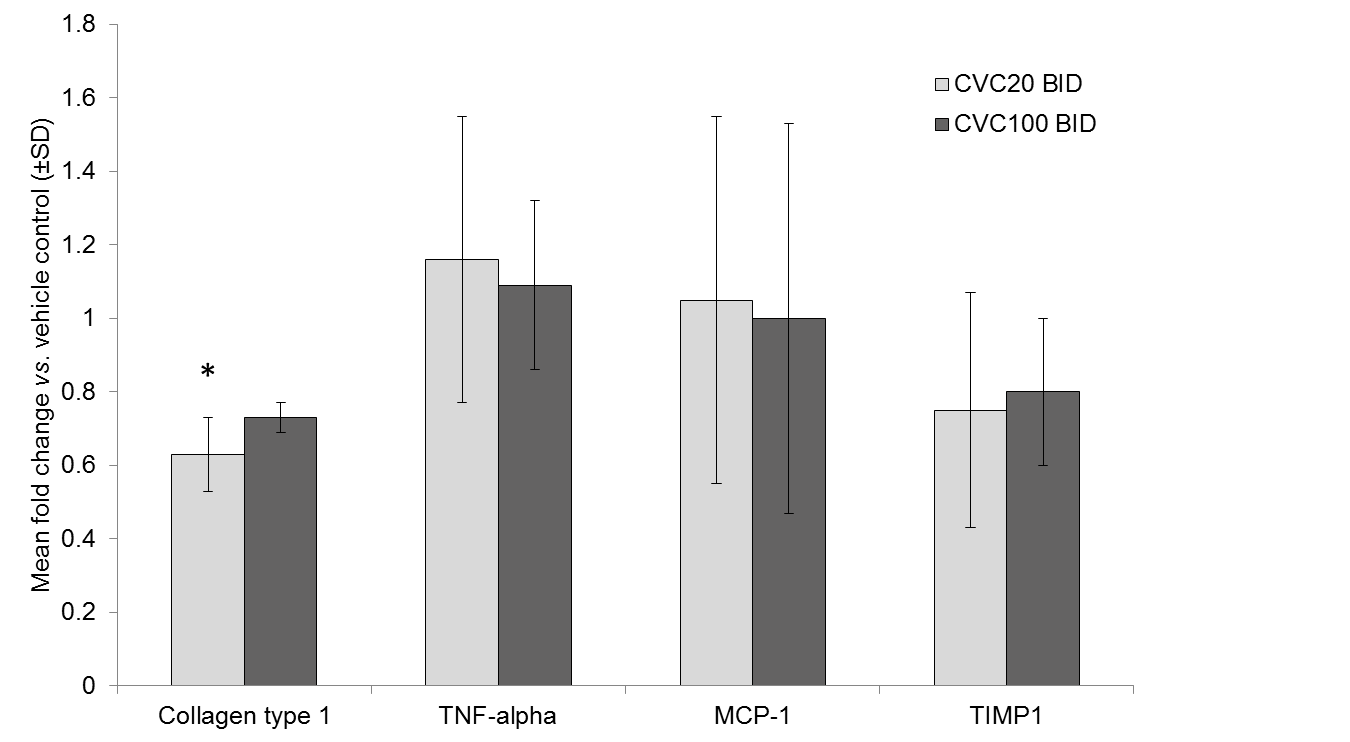

Supplement: S7 Fig — *p < 0.05 vs. vehicle control; BID, twice daily; CVC, cenicriviroc; MCP-1, monocyte chemotactic protein-1; NASH, non-alcoholic steatohepatitis; SD, standard deviation; TIMP, tissue inhibitor of metalloproteinase; TNF, tumor necrosis factor. (TIF) [file pone.0158156.s009.tif]

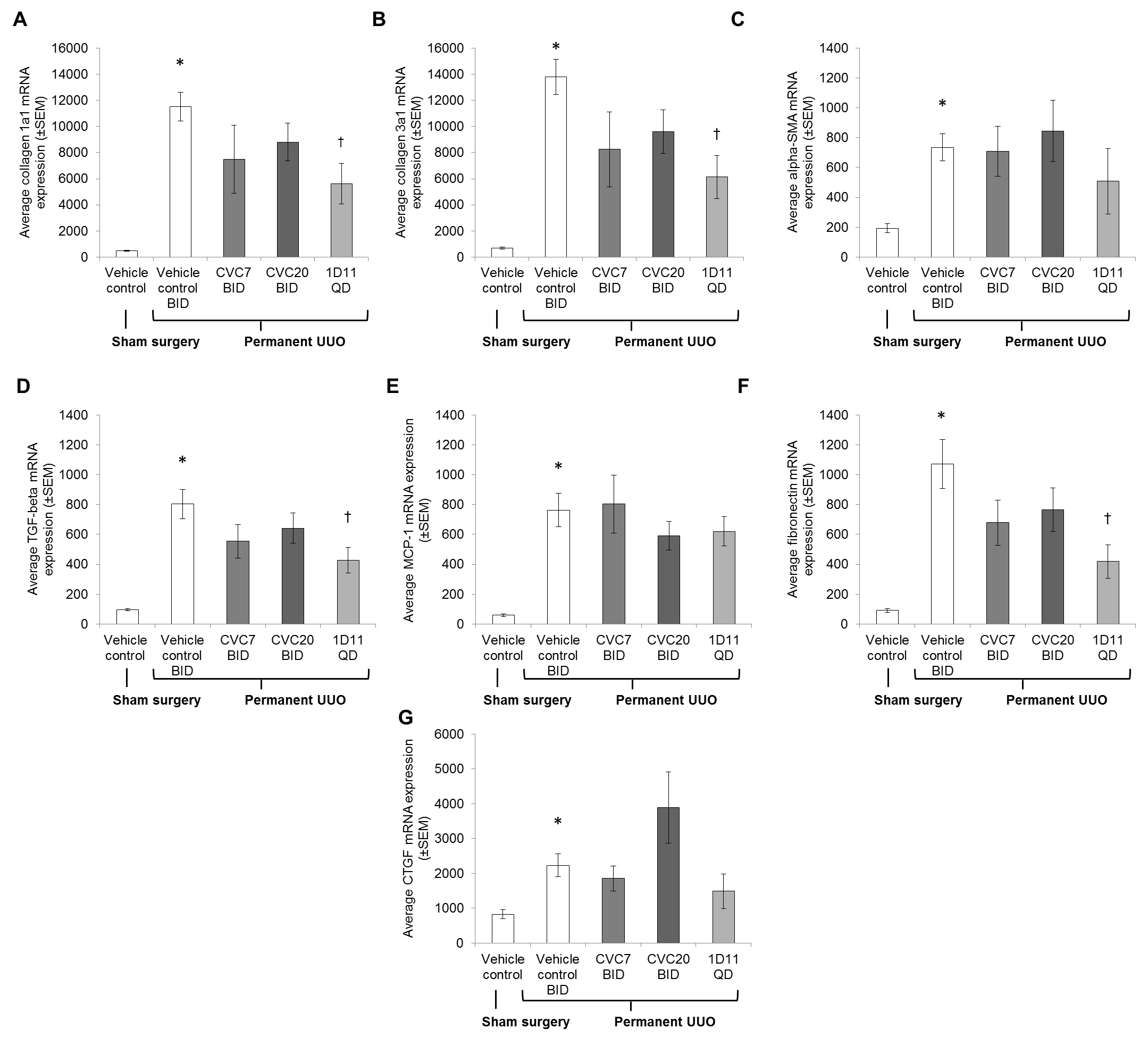

Supplement: S8 Fig — (A) Collagen 1a1; (B) Collagen 3a1; (C) alpha-SMA; (D) TGF-beta; (E) MCP-1; (F) Fibronectin; (G) CTFG. *p < 0.05 vs. sham control; †p < 0.05 vs. UUO control; BID, twice daily; CTFG, connective tissue growth factor; CVC, cenicriviroc; MCP-1, monocyte chemotactic protein-1; QD, once daily; SEM, standard error of the mean; alpha-SMA, alpha-smooth muscle actin; TGF-beta, transforming growth factor-beta; UUO, unilateral ureter obstruction. (TIF) [file pone.0158156.s010.tif]
